# Implementation of the CAPABLE Program Plus Critical Home Repairs Among Older Veterans

**DOI:** 10.1093/geroni/igaf122.4137

**Published:** 2025-12-31

**Authors:** Connor Mitchell, Laura Ellen Ashcraft, Amanda Peeples, Octavia Goodman, Robert Burke, Allyson Evelyn-Gustave, Sarah Szanton, Rebecca Brown

**Affiliations:** Department of Veterans Affairs, Philadelphia, Pennsylvania, United States; University of Pennsylvania, Philadelphia, Pennsylvania, United States; Veterans Health Administration, Philadelphia, Pennsylvania, United States; Corporal Michael J. Crescenz VAMC | U.S. Department of Veterans Affairs, Philadelphia, Pennsylvania, United States; Center for Healthcare Evaluation, Research, and Promotion, Philadelphia, Pennsylvania, United States; Johns Hopkins School of Nursing, Johns Hopkins School of Nursing, Maryland, United States; Johns Hopkins University, Baltimore, Maryland, United States; University of Pennsylvania, Philadelphia, Pennsylvania, United States

## Abstract

Effective programs are needed to facilitate aging in place, particularly among the growing number of older adults with functional decline. The Community Aging in Place-Advancing Better Living for Elders (CAPABLE) intervention, delivered by an interdisciplinary team, helps low-income, community-dwelling older adults to address causes of disability at home. We tested the implementation of CAPABLE among older Veterans in The Safer Aging Though Geriatrics-Informed Evidence-Based Practices Quality Enhancement Research Initiative hybrid type III effectiveness-implementation trial. We adapted CAPABLE to fit the Veterans Affairs (VA) context (CAPABLE-VA) and provided one year of implementation facilitation (e.g., training, implementation team meetings) to sites. Implementation outcomes were measured using the RE-AIM framework, effectiveness outcomes (general health status, ADLs, IADLs, falls self-efficacy, depressive symptoms, pain) using pre- and post-intervention assessments, and qualitative outcomes through post-intervention interviews using the PRISM framework. Four sites enrolled 30 Veterans: 53% (n = 16) received the full clinical dose of CAPABLE-VA; 90% (n = 27) received home modifications (e.g., grab bars, walk-in showers, entryway ramps); and 73% (n = 22) received critical home repairs (e.g. repairing or replacing plumbing, flooring, staircases). Veterans reported significant improvements in overall health status (p < 0.001), ADL performance (p = 0.002), depressive symptoms (p = 0.03), and pain (p = 0.01). Interviews with Veterans and staff indicated that CAPABLE-VA made homes safer and aging in place easier, though the program’s administrative burden was noted. These findings underscore the need to pair CAPABLE with critical home repairs for low-income older Veterans, while highlighting the challenges of reaching Veterans at scale with a complex, multi-component intervention within the VA.

